# Inheritance of central neuroanatomy and physiology related to pheromone preference in the male European corn borer

**DOI:** 10.1186/1471-2148-10-286

**Published:** 2010-09-16

**Authors:** Zsolt Kárpáti, Shannon Olsson, Bill S Hansson, Teun Dekker

**Affiliations:** 1 Max Planck Institute for Chemical Ecology, Department of Evolutionary Neuroethology Hans-Knoell-Strasse 8, D-07745 Jena, Germany; 2 Plant Protection Institute of Hungarian Academy of Sciences, PO Box 102, H-1525, Budapest, Hungary; 3 Division of Chemical Ecology, Swedish University of Agricultural Sciences, PO Box 44, SE-230 53, Sweden

## Abstract

**Background:**

The European corn borer (ECB), *Ostrinia nubilalis*, is a textbook example of pheromone polymorphism. Males of the two strains (Z and E) prefer opposite ratios of the two pheromone components, Z11- and E11-tetradecenyl acetate, with a sex-linked factor underlying this difference in preference. The male antennal lobes of the two strains contain a pheromone sensitive macroglomerular complex (MGC) that is identical in morphology, but reversed in functional topology. However, hybrids prefer intermediate ratios. How a topological arrangement of two glomeruli can accommodate for an intermediate preference was unclear. Therefore we studied the neurophysiology of hybrids and paternal backcrosses to see which factors correlated with male behavior.

**Results:**

Projection neuron (PN) recordings and stainings in hybrids and backcrosses show a dominance of the E-type MGC topology, notwithstanding their intermediate preference. Apparently, the topological arrangement of glomeruli does not directly dictate preference. However, two other factors did correlated very well with preference. First, volumetric measurements of MGC glomeruli demonstrate that, whereas in the parental strains the medial MGC glomerulus is more than 2 times larger than the lateral, in hybrids they are intermediate between the parents, *i.e*. equally sized. Paternal backcrosses showed that the volume ratio is sex-linked and co-dominant. Second, we measured the summed potential difference of the antennae in response to pheromone stimulation using electroantennogram recordings (EAG). Z-strain antennae responded 2.5 times stronger to Z11 than to E11-14:OAc, whereas in E-strain antennae the ratio was approximately equal. Hybrid responses were intermediate to the parents, and also here the antennal response of the paternal backcrosses followed a pattern similar to the behavioral phenotype. We found no differences in frequency and types of projection and local interneurons encountered between the two strains and their hybrids.

**Conclusions:**

Male pheromone preference in the ECB strains serves as a strong prezygotic reproductive isolation mechanism, and has contributed to population divergence in the field. Our results demonstrate that male pheromone preference is not directly affected by the topological arrangement of olfactory glomeruli itself, but that male preference may instead be mediated by an antennal factor, which causes the MGC glomeruli to be differentially sized. We postulate that this factor affects readout of blend information from the MGC. The results are an illustration of how pheromone preference may be 'spelled out' in the ALs, and how evolution may modulate this.

## Background

Moth pheromone communication offers a unique opportunity to study the evolution of behavior and its olfactory processing correlates. The organization of the olfactory circuitry is relatively simple and behavioral responses to pheromones are generally robust. The European corn borer (ECB; *Ostrinia nubilalis*; Lepidoptera: Pyralidae) in particular, has been a textbook example of pheromone evolution. It has a distinct pheromone polymorphism in natural populations, consisting of two strains, which produce and prefer opposite ratios of the two component pheromone blend. The Z-strain produces and prefers a 97:3 blend of (*Z*)/(*E*)-11-tetradecenyl acetate (Z11-14:OAc/E11-14:OAc), whereas the E-strain produces an approximately opposite ratio (1:99 Z:E) [1,2]. Both strains have an interspecific behavioral antagonist, (*Z*)-9-tetradecenyl acetate (Z9-14:OAc) [3]. Female hybrids (ZxE and ExZ) produce an intermediate pheromone blend (35:65 Z:E) and hybrid males respond preferentially to such blends [4,5]. Where the two strains occur in sympatry, hybrids are found in low frequency, between 5-15% [6]. Although other factors (seasonal voltinism and circadian rhythm in particular) have been recognized as reproductive isolation barriers in this species, the absolute strength of male pheromone preference was found to be the strongest (summarized in [7]).

**Figure 1:**
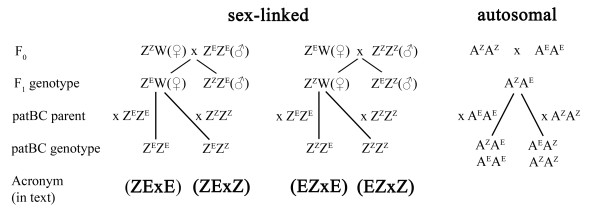
**Expected genotypes of hybrids and backcrosses assuming either sex linkage or autosomal inheritance**. The large Z and W represent the sex chromosomes, A the autosomal chromosomes, whereas in the superscript Z and E represent alleles from respective the pheromone strain. patBC paternal backcrosses (crossing each of the hybrid females with either E-strain (ZExE, EZxE) or Z-strain males (ZExZ, EZxZ)). Note that in Lepidoptera females are the heterogametic sex. This means that paternal crosses, i.e., a female hybrid crosses with a male pure strain, in the case of a sex-linked factor will result in single phenotype offspring only with respect to that factor (either Z, hybrid, or E phenotypes).

The genetic factors responsible for the major pheromone differences between the strains exhibit simple Mendelian inheritance. Female sex pheromone production is controlled primarily by a single autosomal factor [8,9], whereas, male behavioral preference is controlled by a sex-linked factor [4,9-12], this factor being a single gene or a couple of closely-linked genes. Offspring of paternal backcrosses of hybrids (a backcross of a female hybrid with a male pure strain individual) consist of single pheromone phenotypes, i.e., Z, E or hybrid responders (see also Figure 1). In contrast, maternal backcrosses (a cross between a male hybrid and a female pure strain) result in a 1:1 ratio of hybrid and pure strain responders.

The question thus is what factor determines such a major shift in preference. In a previous study we demonstrated how the difference in male preference is correlated with differences in wiring of olfactory input and output neurons in the antennal lobe (AL), which is the first olfactory relay center in the insect brain [13]. The macroglomerular complex (MGC), which is a specialized subgroup of glomeruli in the male AL responsible for the pheromone detection, is morphologically similar in the two strains and consists of two major compartments, a large, medial compartment folded around a smaller, lateral one. Physiological and morphological analyses revealed that the major pheromone component-specific olfactory sensory neurons (OSNs) and PNs arborize in the medial MGC glomerulus in both strains, whereas those sensitive to the minor pheromone component arborized in the lateral one. In other words, the two strains have an indistinguishable MGC morphology, but a reversed functional topology.

However, notwithstanding the intuitive attractiveness of explaining a reversal in male behavioral preference through a swap in functional topology, how the hybrid males' intermediate preference can be accommodated in such a circuit is unclear. We therefore studied the neurophysiology of pheromone preference in this species, its F_1 _hybrids (ExZ and ZxE) and paternal backcrosses (EZxZ, EZxE, ZExE, ZExZ) to find factors in the olfactory circuitry that are sex-linked and correlate with the male behavioral preference reported in other work [5], [12-14]. Paternal backcrosses *i.e*., crosses between a hybrid female and a parental male, are the most decisive, as under sex-linkage the offspring of each cross is expected to have a uniform genotype with respect to that factor (Figure 1).

## Results

Here we present 1) the functional topology of the macroglomerular complex, 2) the response characteristics of projection neurons (PNs) and local interneurons (LNs), 3) the antennal lobe neuroanatomy and 4) the gross antennal response of parental strain, hybrid and paternal backcross males of *O. nubilalis *in order to elucidate the pattern of inheritance of these factors and its correlation with male behavioral preference.

### 1. Functional topology of the MGC in hybrids and backcrosses

**Figure 2:**
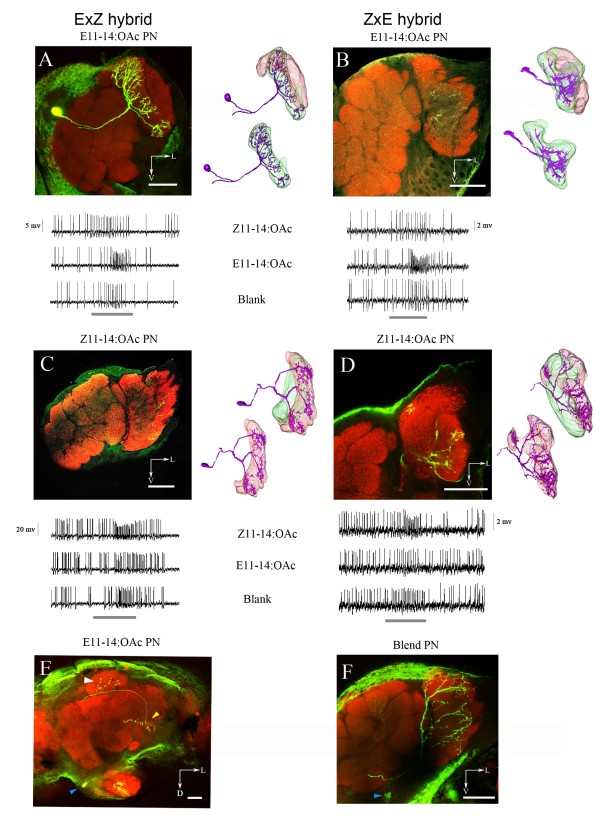
**Dendritic arborization of the projection neurons (PNs) in the macroglomerular complex (MGC) of *O. nubilalis *F_1 _hybrids (ExZ, ZxE)**. Neurobiotin-filled PNs (green) in a α-synapsin-labeled antennal lobe (red). Left panels: confocal stack through a portion of the antennal lobe with part of the neuron visible. Because several confocal sections were overlaid to visualize a large part of the neuron, the otherwise sharp glomerular delineations are somewhat blurred. Right panels: three-dimensional reconstruction of the two large MGC glomeruli (lateral, red; medial, green) and dendritic arborization of the PN (violet). Lower panels: intracellular recording trace of the PN in the upper panel. Stimulation time, 500 ms (grey scale bars). (A) The MGC of a ExZ hybrid male displaying an E11-14:OAc-specific PN with exclusive arborizations in the medial glomerulus. Stimulation dose, 1 ng. (B) The MGC of an ZxE hybrid male displaying an E11-14:OAc-specific PN with exclusive arborizations in the medial glomerulus. Stimulation dose, 10 ng. (C) The MGC of an ExZ hybrid male displaying a Z11-14:OAc-specific PN with exclusive arborizations in the lateral glomerulus. Stimulation dose, 1 ng. (D) The MGC of a ZxE hybrid male displaying a Z11-14:OAc-specific PN with exclusive arborizations in the lateral glomerulus. Stimulation dose, 10 ng. (E) Axonal arborization of the E11-14:OAc-specific PN in the calyces of the mushroom body and in the lateral horn of the protocerebrum. (F) Pheromone blend-specific PN arborization in both large MGC glomeruli, responding only to a 50:50 blend of the Z11-14:OAc and E11-14:OAc. Scale bars in the confocal images, 50 μm.

Antennal lobe neurons in males of EZ and ZE hybrids were characterized. Several classes of neurons were found, including specific, blend and generalist neurons. We were especially interested in projection neurons (PNs) sensitive to only one of the two pheromone components. Of a total of 134 attempted PN fills only 20 (15%) were successful. Component-specific PNs had dendritic arborizations in only one glomerulus, and had their somata in the medial cell cluster (Figure 2A). Axons projected through the inner antenno-cerebral tract (IACT) to the mushroom body calyx and the lateral horn of the protocerebrum (Figure 2E). Blend neurons arborized in both MGC glomeruli, had their cell bodies in the lateral cell cluster (Figure 2F), and projected through the outer antenno-cerebral tract (OACT).

In both hybrids (ZE and EZ) the E11-14:OAc specific PNs arborized without exception in the medial MGC glomerulus (Figure 2A, B), whereas Z11-14:OAc specific PNs arborized in the lateral glomerulus (Figure 2C, D). The PNs responding only to the antagonist (Z9-14:OAc) had dendritic arborization in the posterior glomerulus in both hybrids (n = 3). Apparently, both hybrids (ZE and EZ) have an E-strain functional topology.

**Figure 3:**
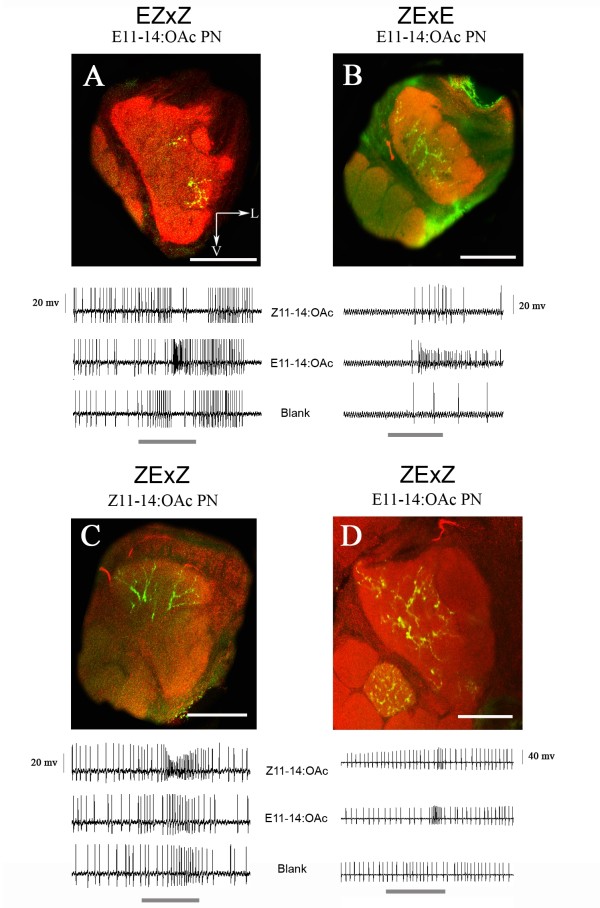
**Dendritic arborization of projection neurons (PNs) in the macroglomerular complex (MGC) in *O. nubilalis *paternal backcrosses**. Other details in Figure 2. (A) E11-14:OAc specific PN in EZxZ males displaying arborizations in the lateral MGC glomerulus only. (B) E11-14:OAc specific PN dendrites in ZExE males arborizing in the medial MGC glomerulus only. (C) Z11-14:OAc-specific PN dendrites arborized exclusively in the lateral MGC glomerulus of the ZExZ paternal backcross male. (D) E11-14:OAc specific PN in ZExZ males with dendritic arbors in the medial MGC glomerulus.

In EZxZ backcross males, dendrites of Z11-14:OAc specific PNs arborized in the medial glomerulus of the MGC, whereas E11-14:OAc specific PN arborized in the lateral glomerulus, similar to the Z-strain (Figure 3A). However, in male ZExE, ZExZ, EZxE backcrosses Z11-14:OAc sensitive PNs arborized in the lateral MGC glomerulus and the E11-14:OAc sensitive in the medial one, an arrangement similar to the E-strain and hybrids (Figure 3B, C, D). The functional topology matches a pattern of sex-linked inheritance with an E-type dominance. Paternal backcrosses to the Z-strain would under autosomal inheritance result in 50% hybrid (E phenotype) and 50% Z phenotypes (Figure 1). Under autosomal inheritance the probability to find 2 times a Z phenotype in the EZxZ cross (2 fills, Table 1) and 3 times a E phenotype in the ZExZ cross (3 fills, see Table 1) is 0.5^5 ^= 0.03.

**Table 1:** Number of recordings and fills from projection neurons in *O. nubilalis *hybrid and backcross males.

	PN	hybrids		backcrosses			
		**ZxE**	**ExZ**	**ZExE**	**ZExZ**	**EZxZ**	**EZxE**

# recordings	Z11	13	22	16	5	13	3
	E11	9	20	17	9	7	4
	blend	19	15	1	1	2	2
# fills	Z11	10	17	15	5	13	3
	E11	6	10	16	9	6	4
	blend	3	12	0	1	2	2
# successful fills	Z11	3	2	1	2	1	1
	E11	2	3	1	1	1	1
	blend	1	0	0	0	0	0

Z11, Z11-14:OAc; E11, E11-14:OAc; blend, blend specialist or blend generalist neuron responding specifically only to one of the blend or to all blends.

### 2. Physiological characterization of PNs and LNs

**Figure 4:**
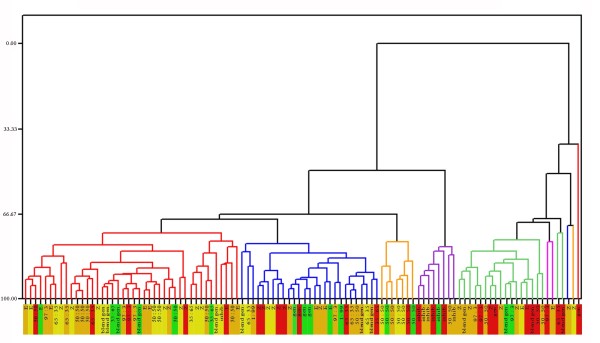
**Complete linkage and euclidean distance cluster analysis of physiologically typed antennal lobe interneurons**. Stimulus dose: 10 ng. Red box, Z-strain; green box, E-strain; orange box, EZ hybrid; yellow box, ZE hybrid. E, E11-14:OAc component-specific PN; Z, Z11-14:OAc component-specific PN; 97:3, 1:99, 65:35, 35:65, 50:50, blend-specific PN responding to the corresponding blend; blend gen., blend generalist PN; gen., generalist PN.

The functional topology of hybrid and backcross males did not correlate with the male behavioral preference. Therefore, we investigated if the response characteristics of the population of PNs and LNs in EZ and ZE hybrids were different compared to the parental strains. Intracellular recording, without neuronal fills were obtained from 26 Z-strain, 16 E-strain, 26 ZE and 40 EZ hybrid males. Each neuron's selectivity was characterized by its response to the two pure pheromone components and blends thereof (97:3, 65:35, 50:50, 35:65, 1:99 Z:E). Neurons in both hybrids and paternal strains were classified according to five types described in an earlier study [15]: 1. component-specific PNs, responding to either Z11-14:OAc or E11-14:OAc, 2. pheromone-blend generalist PNs, responding to all blends, 3. pheromone-blend specific PNs, responding with higher spike frequency to one of the blends, 4. Generalist PNs, responding to all stimuli, and 5. LNs responding with inhibition to all components and blends (Figure 4). The sensitivity of different neurons to the same stimulus was variable. In some cases we found very sensitive and specific PNs responding to as low as a 10 pg dose. The dendrographic tree based on cluster analysis shows that the inhibitory LNs, the 50:50 blend PNs, the Z sensitive PNs and blend PNs cluster separately, but within the same cluster neurons from pure strains and hybrids are interspersed. In other words, we did not find evidence for clusters of PNs specific or more frequent in hybrids.

### 3. Neuroanatomy of the MGC

**Figure 5:**
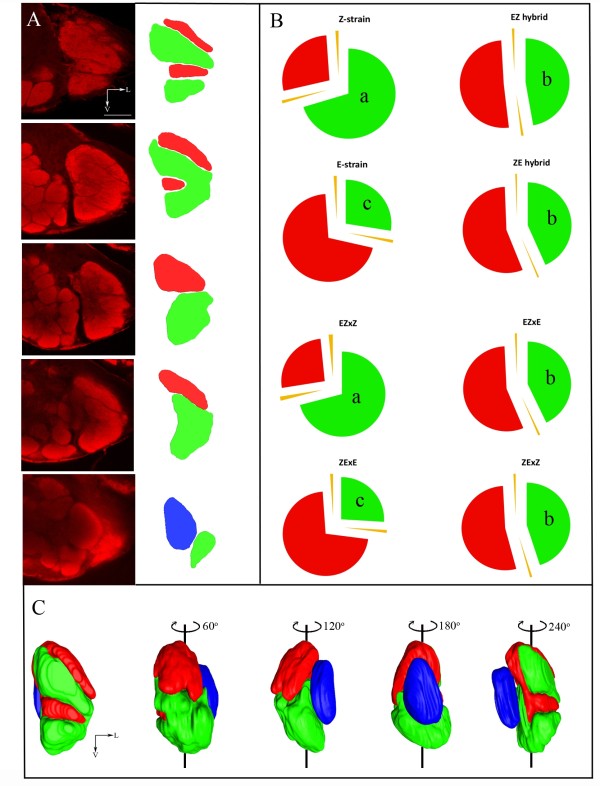
**Structure and size of MGC glomeruli of *O. nubilalis *Z-, E-strain, F_1 _hybrid and paternal backcross males**. (A) Left panels, different confocal sections from anterior to posterior of a F_1 _hybrid male (ExZ) antennal lobe. Right panels, delineations of a confocal section, red, lateral; green, medial; blue, posterior MGC glomeruli. (B) Volumetric measurement of the MGCs of parental strains, hybrids and paternal backcrosses (n = 6). Red, size of the lateral; green, size of the medial compartment; yellow, standard error. Lower case lettering, significance differences (ANOVA followed by a Tukey's HSD post hoc test; P < 0.05). (C) Three-dimensional reconstruction of the MGC depicted in A, rotated along the dorso-ventral axis. Scale bars in the confocal images, 50 μm.

Work on *Drosophila *demonstrated the importance of glomerular size in odor blend preference [16]. We therefore verified if any such correlation would exist in the MGC *of O. nubilalis*. To estimate the volume ratio we reconstructed MGCs of Z-, E-strain, ZxE, ExZ hybrid and paternal backcrosses (ZExE, ZExZ, EZxE, EZxZ). Both Z- and E-strain males had a similar size ratio of the medial:lateral glomerulus (± 70:30). However, in males of both hybrids the ratio of the two large MGC glomeruli was intermediate to the parental strains (ZE hybrid: 56:44 medial/lateral, EZ hybrid 52:48 medial/lateral; Figure 5B).

The neuroanatomical data of the backcrosses followed a pattern predicted under sex-linkage. The volume ratio of EZxZ and ZExE backcross males, which prefer a Z- or E-blend, respectively [5], was similar to the Z- and E-strain (± 73:27 medial:lateral), whereas in the other two backcrosses (ZExZ, EZxE), which both prefer intermediate ratios, it was intermediate (± 54:46 medial/lateral), similar to F_1 _hybrids (Figure 5B).

### 4. Electroantennogram

**Figure 6:**
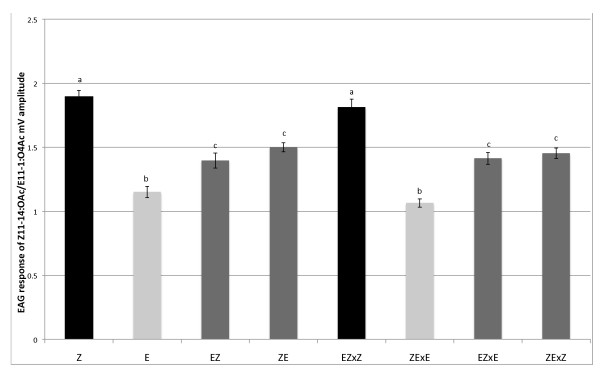
**EAG response of parental strains, hybrids and backcross males**. Y-axes: Relative response of Z11-14:OAc/E11-14:OAc. Statistical differences between measurements, for each group, are shown by lower case lettering (ANOVA followed by a Tukey's HSD post hoc test; P < 0.05).

Glomerular size is, in part, a function of the number of specific OSNs in the antennae projecting their axons into the AL glomeruli. The number of OSNs in turn is partially correlated with the overall antennal response to a given key ligand of this OSN [16,17]. We therefore measured the EAG depolarization of males of parental strains, hybrids and backcrosses in response to Z11-14:OAc and E11-14:OAc, and expressed it as a proportion of Z11-14:OAc: E11-14:OAc. Whereas Z-strain male responded more strongly to Z11-14:OAc than to E11-14:OAc (n = 15), responses in the E-strain to both isomers were similar (n = 15, Figure 6). We also measured the EAG responses of the American Z- and E-strain and found no differences compared to the European strains (data not shown). In both F_1 _hybrids (ZxE, n = 33; ExZ, n = 18) the relative response was intermediate to the Z-, and E-strain. EZxZ backcross males responded to Z11-14:OAc with higher amplitude, similar to the Z-strain (n = 60), the ZExE backcross males responded like the E-strain (n = 19), whereas the two other backcrosses (EZxE, n = 48; ZExZ, n = 86) had EAG amplitude ratios similar to F_1 _hybrids. The EAG response thus followed a pattern congruent with the volume ratio of the two major MGC glomeruli, and is consistent with sex-linkage (Figure 6).

## Discussion

Lepidoptera commonly use pheromones in sexual communication. Numerous examples illustrate the enormous diversity and evolutionary divergence of pheromone production and preference in this species rich taxon. Yet, the neurogenetics underlying male preference have been studied in only few cases. In these cases shifts of pheromone preference have been linked to shifts in detection in the periphery, *i.e*., the antennae.

In *Trichoplusia ni*, directed laboratory selection for male preference to a mutant pheromone blend resulted in a dramatic desensitization of OSNs responding to a minor component, which is produced in excessive amounts by hybrid females [18,19]. In *Agrotis segetum*, pheromone production in populations across Europe and Africa showed differences in composition of pheromone components, which was paralleled by changes in sensillar distribution in antennal branches [20]. In *Heliothis virescens *and *H. subflexa*, which use different minor pheromone components in their blend, differences in male preference were explained in part by subtle shifts in the tuning curve of OSNs [21,22], and in part by a postulated change in interpretation of glomerular output in higher brain centers [23]. Finally, *Ostrinia furnacalis *and *O. nubilalis *males are incidentally attracted to each other's pheromone blend. These 'rare' males exhibited minor alterations in the response profile of the pheromone sensitive OSNs, such that the resulting input pattern could resemble the blend of their cognate species [24,25].

Although male pheromone preference in the ECB, *Ostrinia nubilalis*, most significantly contributes to reproductive isolation and speciation between its two strains, the neurogenetic basis is unresolved. Here we found in the two strains of ECB, their hybrids, and backcrosses neurophysiological correlates of male pheromone preference reported in earlier studies [5].

### Functional topology of the MGC in hybrids and backcrosses

Previously, in a detailed study on the neuroanatomy and physiology of the MGC in *O. nubilalis *[13] we demonstrated that the two strains have an identical MGC neuroanatomy. However, OSNs and PNs sensitive to the major pheromone component arborized in both strains in the medial glomerulus, *i.e*., the MGC of the two strains are functionally mirror images of each other. Although this correlates well with a reversal of preference between the parental strains, intermediate preferences, such as found in hybrids, are not easily explained by such a switch.

In this study we show that the functional topology of MGCs of both F_1 _hybrids (ZxE, ExZ) is E-dominant, in spite of their intermediate preference [4,26]. The functional topology may be a determined through axonal diameter, which seems E-dominant and sex-linked in recent work [27]. In a striking parallel, hybrids of two heliothine species (*H. virescens *and *H. subflexa*) displayed a functional topology that is most reminiscent of one of the species (*H. subflexa*), in spite of their more intermediate behavioral preference for pheromones. Be it as it may, the topological phenotype in *O. nubilalis *does not match the behavioral phenotype, and therefore does not explain male moth preference.

### Physiological characterization of PNs and LNs

Although the topological arrangement of MGC glomeruli in hybrids is of an E-type and cannot underlie hybrid preference, it is conceivable that the shift in behavioral preference is mediated through a shift in the tuning curve of these glomeruli, as measured through either component-specific or blend PNs, and would induce an intermediate preference. Such subtle changes in the tuning or the innervation of specific MGC glomeruli correlated with differences in male pheromone preference between *Heliothis virescens *and *H. subflexa *and their hybrids (see above, [28]). If true, one would expect either a numerical over- or underrepresentation of certain PN and LNs types in hybrids, or unique clusters of neurons with different response properties arising in hybrids. Our cluster analysis did not reveal a segregation of pure strain and hybrid PNs and LNs with respect to either their occurrence or their tuning curve. However, since we only sampled a subset of neurons, more extensive recordings may give rise to such clusters.

### Volume of MGC glomeruli and OSN input

Recently, it was shown that glomerular size correlated with odor preference in the fruit fly, *D. sechellia *(Ibba I, Angioy AM, Hansson BS, Dekker T: Macroglomeruli tuned to fruit odors radically change blend preference in *Drosophila*, submitted) [16]. An overrepresentation of certain OSNs resulted in fruit-odor-tuned macroglomeruli, and caused increased attraction to key ligands of OSNs innervating these glomeruli. This is not necessarily intuitive, as one could equally argue that larger glomeruli could induce repulsion and hypersensitivity [29]. While the above finding concerned fruit flies and general odors, we conjectured that similar coding principles might govern pheromone coding. Here we found that, in fact, the relative size of *O. nubilalis *MGC glomeruli correlated well with male preference and sex-linkage. Although an increase in number of OSN and a concomitant increase in glomerular size have been implied in lowering detection thresholds, here we postulate that *within *the MGC the relative size of glomeruli fine tunes male blend preference and specificity. It is interesting to note that in Lepidoptera, without exception, the largest MGC glomerulus is tuned to the major pheromone component [30-32].

Several factors contribute to the size of a glomerulus, including the number or the arbor size of neurons. In *Drosophila *a strong correlation was found between number of OSNs and glomerular volume [16], as well as between dendritic diameter and glomerular size [29]. With respect to the latter, the observation that OSNs in hybrids have an intermediate diameter [33] and that spike amplitude appears sex-linked seem fitting. Although the correlation between EAG response (see later on) and glomerular size is strong, both being sex-linked and intermediate in hybrids, it is possible that other neurons at more central level also contribute to the difference in glomerular size. For example, Scott et al. found that plasticity in the glomerular size of the visual system of *Drosophila *was influenced by sensory neuron input and LNs, but not by PNs [34,35].

Of interest is also the fact that the size ratio of MGC glomeruli is not linear with male behavioral preference, *i.e*., the volume ratio in parental strains is 72:28, whereas the male preference follows roughly female production, *i.e*., 97:3 (1:99 for the E strain). If volume ratio is a determinant in male preference, it would imply that relatively small shifts in size can cause disproportional shifts in preference. Such a disproportional behavioral shift was also observed in the aforementioned study on *Drosophila *flies (Ibba I, Angioy AM, Hansson BS, Dekker T: Macroglomeruli tuned to fruit odors radically change blend preference in *Drosophila*, submitted) [16]. Further studies are needed to address the question of what modulates glomerular size, and how this affects blend readout in insects. The importance of factors that underlie voluminar shifts is underlined by other studies where shifts were recorded during an insect's life span to due to changes in social status in *Apis mellifera *(e.g. [36]), exposure (in *Drosophila*, [37,38]), or adult development (in *Manduca sexta *[39]).

### Correlation between the antennal response and pheromone preference

Glomerular size is partially determined by the number of OSNs arborizing in a glomerulus [16]. We therefore measured the response of whole antennal preparations to both pheromone components, as the sum potential difference in response to odors measured through EAGs are thought to reflect (in part) OSN abundance [16,17,40]. The results of parental, hybrid and backcross males clearly demonstrate that the antennal response is sex-linked. The antennal response supports the idea that the response factor may be located upstream from the antennal lobes, in the antennae [13].

Furthermore, the results render support to the idea that the number and/or axonal diameter of OSNs innervating the E and Z glomerulus differ between the two strains. In the Z-strain the response to Z11 was substantially larger than to E11. As the relative sum potential is thought to in part reflect the relative number of OSNs tuned to the compound [16,17,40], this could indicate a prevalence of Z11 over E11 OSNs in the Z-strain. Previous physiological work in *O. nubilalis *has demonstrated the existence of three types of sensilla trichodea housing OSNs sensitive to pheromone components. Type A and B house one OSN sensitive to Z11 and one to E11. In contrast, type C, which is rather rare [41] has a single OSN responding to the main pheromone component [9,14,41], although a precise distribution and frequency map across the antenna is lacking. This casts the question of to what extend rare sensilla and their associated neurons could affect the EAG ratio. The matter is further complicated by the fact that in the E-strain the EAG response to Z11 was surprisingly similar to E11. This either implies that the ratio of E11 versus Z11 sensitive OSNs in the E-strain is similar, or that the E11 receptors are more broadly tuned (responding also to Z11) than the Z11 receptors (responding less or not at all to E11, [19,42]), or both. However, identification and deorphanization of *O. nubilalis *pheromone receptors underlying the OSN response is required to substantiate this.

Although it is uncertain what precisely causes the differential EAG sum potentials between pheromone components and strains, our EAG results are cohesive with the differences found in volume ratio of MGC glomeruli, and support the idea that the shift in preference may be located in the periphery. However, since the 'genetic factor' is not necessarily one single gene, and could be a couple of closely-linked genes, mechanisms downstream from the antennae may also contribute to the shift in pheromone preference in the ECB.

## Conclusions

Pheromone preference of male ECB is a strong mechanism in prezygotic reproductive isolation and incipient speciation [7], although its proximate mechanisms are obscure. Here we demonstrate that pheromone preference in the European corn borer is not directly correlated with the position of the MGC glomeruli, but rather by a factor correlated with MGC glomerular size and antennal response. This may imply that blend information is read out from the antennal lobes in such a way that shifts in glomerular size can dramatically alter blend preference. This work contributes to understanding how evolution can act on the olfactory circuitry to shape pheromone preference, an important contributor to reproductive isolation and speciation.

## Methods

### Insects

The adult Z-strain was derived from a colony established from a cornfield collection in Kéty town, county of Tolna, Hungary in 2004. The E-strain colony was established from a 2005 collection of larvae from maize stems by Smiljana Tomse (Agriculture and Forestry Institute, Novo Mesto, Slovenia). All strains, hybrids and paternal backcrosses were reared on a semi-artificial diet [43] at 25°C, RH 70% under L:D = 17:7 photoperiod. The genetic purity of the cultures was monitored by gas chromatographic analysis (GC) of female pheromone production. F_1 _hybrids were produced by crossing Z females with E males (ZE hybrid) and E females with Z males (EZ hybrid). Paternal backcrosses were obtained by crossing each of the hybrid females with either E-strain (ZExE, EZxE) or Z-strain males (ZExZ, EZxZ) (Figure 1). The males had access to a 5% honey water solution. Non-anesthetized male moths of 0-4 days old were used for the experiments. For EAG measurements we also used the American Z- and E-strain (kindly provided by Charlie Linn; Cornell University, New York, USA). However, unless otherwise specified we used the European strains for experiments.

### Intracellular recording and neuronal filling

A male was inserted in a 1 ml plastic pipette tip such that the head protruded from the tip. The head was supported and immobilized with dental wax (Surgident, Miles Inc., USA). The scales on the head and the proboscis were removed. A window was created by cutting the cuticle between the two eyes, through which the antennal lobes were visible. Muscles surrounding the antennal nerve were removed for stable recordings. The preparation was placed in an electrophysiological setup and tilted such that the antennal lobes were visible through the microscope. The preparation was constantly infused with Tucson ringer solution of pH 6.9 containing 8.55 g/l sucrose [44]. A silver reference electrode was inserted into the brain, close to the antennal lobes. A glass recording electrode (Borosilicate glass with filament, ID 0.5 mm, OD 1 mm, Sutter Instrument, Novato, CA, USA) was pulled by using a horizontal flaming/brown micropipette puller (P-97, Sutter Instrument). The tip of the recording glass electrodes were filled with 1% Neurobiotin™ dye (Vector Laboratories, Burlingame, CA, USA), whereas the shaft was filled with 1M KCl solution. The resistance of the electrodes was measured in the extracellular medium of the preparation and was between 100-250 MΩ. Using a micromanipulator the recording electrode was inserted into the antennal lobe from the top, close to the antennal nerve entrance, where the MGC is located. Usually, the most successful recordings were obtained close to the surface of the antennal lobe.

When intracellular contact was established, the ipsilateral antenna was stimulated with the pheromone components (Z11-14:OAc, E11-14:OAc), their blends (50:50, 97:3, 1:99, 65:35, 35:65 Z:E) and the pheromone antagonist (Z9-14:OAc), diluted in redistilled n-hexane and applied on a filter paper (1 × 1 cm, Munktell Filter AB, Falun, Sweden) inside a Pasteur pipette. The purity of the odorants was verified using GC. A hexane blank served as a control. The cartridges were stored in airtight boxes at - 20°C. The single pheromone components and blends were tested at 1 and 10 ng, and in some case 1, 10 and 100 pg. The dose of blends reflects the total quantity of two pheromone components together, e.g. a 10 ng 50:50 Z:E, was prepared with 5 ng Z11-14:OAc and 5 ng E11-14:OAc. During the physiological recordings a constant, charcoal-filtered and humidified airflow (8.3 ml/s) was blown through a glass tube (ID 7 mm) over the ipsilateral antenna. During the 0.5 s stimulation the airflow was 8.3 ml/s. (Stimulus controller: SFC-2/b, Syntech, Kirchzarten, Germany). The stimulation pipette was inserted in the glass tube 20 cm from the antenna. The odor stimuli were presented at 10 s inter-stimulus intervals and started with the 1 ng stimulus dose.

The activity of the neuron before, during and after stimulation was recorded. The recording electrode was connected to a headstage (HS-2A, Axon Instruments, Foster City, CA, USA). The analog signal was amplified (Axonprobe, Axon Instruments), converted to a digital signal (IDAC 4, Syntech) and visualized using AutoSpike 3.7 software (Syntech). Action potentials were counted manually. The response of the neurons was expressed as the number of spikes during a 0.5 s period after stimulus onset minus the spontaneous activity (number of spikes 0.5 s before stimulus onset), and expressed as the number of spikes per s. The response characteristic of the interneurons were analyzed by using cluster analysis (MINITAB 14, Coventry, UK) and presented in dendrograms with complete linkage and euclidean distance. After physiological characterization the neuron was injected iontophoretically with Neurobiotin™ dye by passing 0.8-1.2 nA constant depolarizing current through the recording electrode for 10-15 min. Brains were processed as described under 'neuroanatomical techniques'.

### Neuroanatomical techniques

The heads were fixed for three hours at room temperature in 4% PBS (phosphate buffer saline) buffered (pH: 7.2) formaldehyde solution containing 0.25% Triton X-100 (Tx). The fixed brains were dissected, washed 4 × 10 min in PBS + 0.25% Tx (PBS+Tx), and incubated in PBS+Tx with 3% α-synapsin antibody (Hybridoma, Univ. of Iowa, Iowa, IA, USA) and 3% fluorescein Avidin (NeutrAvidin, Oregon Green 488 conjugate, Invitrogen, Eugene, Oregon, USA) overnight on a horizontal rotator at RT. For better resolution 3% phalloidin Alexa Fluor 546 (Invitrogen) was added. The next day brains were washed 4 × 10 min in PBS+Tx and incubated with 1% α-mouse (goat) Alexa Fluor 546 (Invitrogen) in PBS+Tx three hours on the rotator at room temperature. Finally, the brains were washed 4 × 10 min in PBS+Tx and transferred in Vectashield Hard set (Vector Laboratories, Burlingame, CA, USA) mounting medium.

A confocal microscope (Zeiss LSM 510, Carl Zeiss, Jena, Germany) equipped with a 40 ×, 1.4 oil-immersion DIC objective lens was used to examine the mounted brains. Structures labeled with fluorescein Avidin and Alexa 546 were excited with an Argon (488 nm) and a He/Ne laser (543 nm) respectively, and their fluorescence was detected after passing through a band pass (505-530 nm) and a long pass (560 nm) filter, respectively. The brains were scanned with a 0.9 μm optical sections for detailed imaging. Stacks of 50-200 confocal images were analyzed by scrolling through optical sections to identify the fine structure of the MGC and dendritic arborization of the PNs. The three-dimensional reconstructions, volumetric measurements of the MGCs and visualization of the individual PNs were done using AMIRA 4.0 software (Visage Imaging, Berlin, Germany). In every second optical section the contours of glomeruli were demarcated by hand (i.e., image segmentation) and interpolated. Arcsin transformed data were analyzed using a one-way analysis of variance (ANOVA) followed by a Tukey's HSD post hoc test.

### Electroantennogram (EAG)

Males of Z-, E-strain, their hybrids (ZE, EZ) and paternal backcrosses (ZExZ, ZExE, EZxZ, EZxE) ECB were tested for their antennal olfactory responses to Z11- and E11-14:OAc. A freshly emerged, naïve male antenna was cut at the base and placed between a silver reference and recording electrode with electrically conductive gel (Blågel, Cefar, Lund, Sweden). The electrode holder was connected to a high impedance 10× gain input stage, which was connected directly to the acquisition controller (IDAC-2, Syntech). For the stimulation the pheromone components were diluted in redistilled n-hexane and applied on a filter paper (1 × 1 cm, Munktell Filter AB, Falun, Sweden) inside a Pasteur pipette. The purity of the odorants was verified using GC. Every experimental day the stimuli were prepared freshly. A dose of 500 ng was used for each pheromone components. Each cartridge was stimulated equally much. A hexane blank served as a control. During the measurement charcoal-filtered and humidified air (6.7 ml/s) was delivered continuously through a glass tube (ID 7 mm) over the antennal preparation using a stimulus controller (CS-55, Syntech). The Pasteur pipette, containing stimulus, was inserted into the main airstream 20 cm from the antenna. Stimuli consisted of a 0.5 s block pulse using 6.7 ml/s pulse airflow. Inter-stimulus intervals were 25 ± 5 s. The stimulation order was: Z11-14:OAc, E11-14:OAc, blank, E11-14:OAc, Z11-14:OAc, blank. The signal was visualized using Syntech software (GC/EAD 32 version 4.3 software). The amplitudes of the antennal response were measured and the responses of the same stimuli were averaged and normalized against the averaged blank. The Z11-14:OAc response was divided by E11-14:OAc. Arcsin transformed data were analyzed using a one-way analysis of variance (ANOVA) followed by a Tukey's HSD post hoc test.

## Authors' contributions

ZK participated in the design of the study, carried out the experiments, performed statistical analyses. TD financed, mentored, participated in the design of the study, carried out part of the experiments and wrote the manuscript together with ZK. BSH participated in the design of the study and corrected the manuscript. SO participated in the EAG experiments. The authors read and approved the final version of the manuscript.
